# The anticancer effect of recombinant LukS-PV protein and silver nanoparticles loaded with this protein

**DOI:** 10.1186/s13568-023-01558-3

**Published:** 2023-06-08

**Authors:** Hafizeh Haghighatafshar, Bahram Golestani Eimani, Elham Moazamian, Jafar Amani

**Affiliations:** 1 Department of Microbiology, Faculty of Sciences, Agriculture and Modern Technology, Shiraz Branch, Islamic Azad University, Shiraz, Iran; 2 Department of Biology, Urmia Branch, Islamic Azad University, Urmia, Iran; 3 Applied Microbiology Research Center, Systems Biology and Poisonings Institute, Baqiyatallah University of Medical Sciences, Tehran, Iran

**Keywords:** LukS-PV protein, Silver nanoparticles, Cloning, Expression, Breast cancer, Anticancer activity

## Abstract

LukS-PV is a component of Panton-Valentine leucocidin (PVL) and is secreted by *Staphylococcus aureus*. Silver nanoparticles exhibit considerable potential as anticancer agents and drug delivery systems. Drug delivery is a way to deliver medicinal combinations to achieve a beneficial therapeutic effect. In the current study, recombinant LukS-PV protein-loaded silver nanoparticles were prepared and their cytotoxicity effect was analyzed on human breast cancer cells and human normal embryonic kidneys cells by MTT assay. Apoptosis was investigated by staining with Annexin V/propidium iodide. The recombinant LukS-PV protein-loaded silver nanoparticles showed dose‐dependent cytotoxicity and induced apoptosis in the MCF7 cells and had a lesser effect on HEK293 cells. After 24 h exposure to the recombinant LukS-PV protein-loaded silver nanoparticles (IC50), Annexin V-FITC/PI FCM revealed that 33.2% of MCF7 cells were apoptotic. In conclusion, recombinant LukS-PV protein-loaded silver nanoparticles probably cannot be a better alternative for the targeted healing approaches to cancer therapies. Hence, it is suggested that silver nanoparticles could be utilized as a delivery system for releasing toxins into cancer cells.

## Introduction

Breast cancer is the most common malignancy among women and the second reason for death that accounts for approximately 25% of all cancers among women (Khan et al. 2022; Dubey et al. 2022). Breast cancer in women causes the highest morbidity (Dubey et al. 2022). Patients with metastatic breast cancer have a poor prognosis. Their survival is less than 5 years (Barzaman et al. 2020). Surgery to remove the breast tumor, medicines including anti-estrogen, aromatase inhibitors, anti-angiogenesis, and radiation therapy are standard breast cancer treatments (Wang et al. 2019; Waks and Winer 2019). However, the high toxicity of existing breast cancer chemotherapeutics, development of drug resistance, and subsequent relapses are the major challenges in treating breast cancer patients (Barzaman et al. 2020; Wang et al. 2019) and new approaches are needed.

In recent years, bacterial toxins-based therapeutics have been proposed as a promising approach in cancer treatment due to their specificity and cytotoxicity in binding to target cells (Kramer et al. 2018; Xu et al. 2021; Moazamian et al. 2018). For instance, Botulinum neurotoxin type C is produced by strains of *Clostridium botulinum* and induces apoptosis in differentiated human neuroblastoma cells (SH-SY5Y and SiMa) (Rust et al. 2016). A fragment of azurin (p28) produced by strains of *Pseudomonas aeruginosa* strains displays anticancer and antiangiogenic properties in breast cancer (Baindara and Mandal 2020).

Panton-Valentine leucocidin (PVL) is a pore-forming toxin, composed of two subunits, LukS-PV and LukF-PV. PVL is produced by *Staphylococcus* aureus and induces pores in the membranes of cells such as polymorphonuclear neutrophils (PMNs), monocytes, and macrophages (Nawrotek et al. 2018; Ma et al. 2020). Previous studies demonstrated that the LukS-PV subunit had no pore-forming capability when used alone; nevertheless, this subunit can induce apoptosis and differentiation in different cancer cell lines (Shan et al. 2017; Qiang et al. 2020; Dai et al. 2016).

Nanotechnology is now found to be a novel agent in cancer therapies. Nanoparticles have unique physical and chemical properties such as small sizes, large surface area, high surface reactivity, a high surface-volume ratio also considerable biological effects, such as drug delivery, disease imaging, antibacterial, and anticancer activities (Slavin et al. 2017; Kovács et al. 2022; ud Din et al. 2017; Sim and Wong 2021).

Silver nanoparticles (AgNPs) demonstrate significant potential in the field of medicine, including in drug delivery systems and cancer therapeutics (Ratan et al. 2020; Alahmad et al. 2021). Therefore, they are regarded as potential tools to enhance the effectiveness of therapies. The anticancer activity of chemically synthesized AgNPs has been previously studied. It has also been elucidated that chemically synthesized AgNPs display significant anticancer activity with fewer side effects compared with chemotherapy agents (Yesilot and Aydin 2019; Dinparvar et al. 2020). Therefore, AgNPs could be a novel therapeutic strategy in cancer treatment because they deliver targeting antitumor drugs or toxins to the tumor tissues.

According to the anticancer effects and drug delivery capability of silver nanoparticles and the anticancer effects of bacterial toxins, for instance LukS-PV, recombinant LukS-PV protein-loaded silver nanoparticles may be considered a new composition for cancer therapy with multiple benefits over other therapeutic compositions. In the current study, designing, cloning, and expression of the recombinant LukS-PV gene was carried out in *Escherichia coli*, and purification of this protein was performed. The chemical synthesis of silver nanoparticles using a trisodium citrate reducing agent was reported. Then, recombinant LukS-PV protein-loaded silver nanoparticles (AgNPs + LukS-PV) were characterized by size, Ζ‐potential, loading efficiency, and toxin release. The cytotoxicity effect of recombinant LukS-PV protein, chemically synthesized AgNPs, and AgNPs + LukS-PV on human breast cancer cell line MCF7 and human normal embryonic kidney cell line HEK293 was conducted using the MTT method. Finally, flow cytometry was done to detect cell apoptosis.

## Materials and methods

### Design, cloning, and expression of the LukS-PV coding gene

The DNA sequence of the LukS-PV gene of *Staphylococcus aureus* strain HT20010734 was obtained from the National Center for Biotechnology Institute (NCBI) at http://www.ncbi.nlm.nih.gov (accession number: EU518761.1). The LukS-PV gene was optimized using the web-based program, ATGme at http://atgme.org/?i=1. *Eco*RI and *Hind*III restriction sites were added at the 5′ and 3′ ends, respectively, then 6 His-tagged was added at the 5′ end. Finally, the optimized LukS-PV gene (accession number: OQ435276) attached to the pUC-57 cloning vector was synthesized by ShineGene Company, China. Following this, the LukS-PV gene was subcloned into the PET 28a expression vector. Then the PET 28a + LukS-PV construct was transformed into the competent *E. coli* Rosetta (DE3) cells. Screening of recombinant bacterial colonies was performed via colony PCR by T7 universal primer (forward: TAATACGACTCACTATAGGG; reverse: GCTAGTTATTGCTCAGCGG). The PCR product was analyzed by 1% agarose gel electrophoresis. IPTG (Isopropyl β-d-1-thiogalactopyranoside-Thermo Fisher Scientific, Germany) was used to induce gene expression. Analysis of the samples was carried out using sodium dodecyl sulfate–polyacrylamide gel electrophoresis (SDS-PAGE). Gels were stained by Coomassie Brilliant Blue R-250 (Sigma-Aldrich, Germany).

### Recombinant protein purification

Protein purification was carried out under denaturing conditions using a Ni- NTA column (Thermo Fisher Scientific, Germany). For this purpose, bacterial pellets were resuspended in native buffer (50 mM NaH_2_PO_4_, 300 mM NaCl, 10 mM imidazole, pH 8.0). Ultrasonicator was used to perform the sonication. Inclusion bodies were resuspended in denaturing buffer (100 mM NaH_2_PO_4_, 10 mM Tris–Cl, 8 M urea, pH 8.0). In the next step, the Ni–NTA column was equilibrated with denaturing buffer before sample injection. Then the supernatant was transferred onto the column. The column was washed with 5 ml of washing buffer (100 mM NaH_2_PO_4_, 10 mM Tris–Cl, 8 M Urea, pH 6.3) to remove unbound proteins. Elution buffers (100 mM NaH_2_PO_4_, 10 mM Tris–Cl, 8 M Urea, pH 4.5) were used to elute the His-tagged protein. Finally, the purified protein was subjected to urea removal and refolded by a dialysis bag (12 kDa MWCO) against phosphate‐buffered saline (PBS) (pH 7.5). Samples were run on 12% SDS-PAGE.

### Western blotting analysis

For Western blotting analysis, Tris Buffered Saline + Tween 20 (TBST), transfer buffer, diaminobenzidine solution (DAB), and horseradish peroxidase (HRP) conjugated anti-His-tag at 1:2000 dilution were prepared. First, gels were blotted to polyvinylidene difluoride (PVDF) membrane (Roche) and blocked with 5% skimmed milk powder in TBST for 2 h to prevent non-specific reactions. Then the PVDF membrane was washed three times with TBST buffer and was incubated for an hour in a shaker incubator with HRP conjugated anti-His-tag. Afterward, washing was repeated three times with TBST buffer. Finally, protein bands were detected by diaminobenzidine solution. Protein concentration was determined by the Bradford method (Kielkopf et al. 2020) with bovine serum albumin (BSA) as the standard.

### Chemical synthesis of AgNPs

Chemical synthesis of AgNPs was performed using a tri-sodium citrate reducing agent (Yerragopu et al. 2020). Briefly, 10 mg of silver nitrate (AgNO_3_) was added to 50 ml double distilled water; the solution was heated at 90 °C using a heater stirrer. 5 ml of 1% tri-sodium citrate was added drop by drop to this solution. The reaction was allowed to take place until the color changed to yellow, which showed the formation of AgNPs. Following this, the solution was cooled to room temperature and stirring was continued. Then, 200 μ of 0.3% Polyvinylpyrrolidone (PVP) was added to the solution and it was stirred on a magnetic stirrer for 10 min. The surface morphology was investigated using a Field Emission Scanning Electron Microscope (FE-SEM). The size and zeta potential of AgNPs were measured using dynamic light scattering (DLS) (Nano ZS zeta sizer system; Malvern Instruments).

### LukS-PV protein loading to AgNPs

Different ratios of protein to AgNPs (1:1, 2:1, 3:1, and 4:1) were prepared with a final mass of 2 ml. The solutions were placed on a shaker speed of 180 rpm for 16 h. To determine loading percentage of protein to silver nanoparticles the solutions were centrifuged at 12,000 rpm for 10 min. The supernatant that contains unloaded protein was evaluated by Bradford assay, and loading efficiency was calculated by below equation

$$\mathrm{Loading\,efficiency \%}=\frac{\mathrm{mass\,of\,protein\,used}-\mathrm{protein\,in\,the\,suspension}}{\mathrm{mass\,of\,protein\,used}}\times 100$$

### In vitro release of recombinant LukS-PV protein from AgNPs

For this purpose, the best loading efficiency ratio of recombinant LukS-PV protein to silver nanoparticles was prepared. These solutions were centrifuged at 12,000 rpm for 10 min. The supernatant was decanted and the pellet was resuspended in 1 ml PBS. The tubes were shaken on a shaker incubator at 37 °C and were sampled at time frames 0, 1, 4, 8, 16, 24, 48, 72, 96, and 120 h. Bradford assay determined the release of recombinant LukS-PV protein from AgNPs.

#### Cell culture

The MCF7 cell line and HEK293 cell line were purchased from the cell bank of the Pasteur Institute of Iran. Cells were cultured in DMEM/F12 (Gibco, Germany) supplemented with 10% fetal bovine serum (FBS) (Gibco, Germany) and 1% penicillin/streptomycin. Cells were cultured at 37 °C in a humidified incubator containing 5% CO_2_.

### Cytotoxicity assay

Cytotoxicity was evaluated using an in vitro MTT-based cytotoxicity assay kit (DNAbioTech). Cells were seeded in 96-well plates at a density of 1 × 10^4^ cells/well in a total volume of 100μL. After overnight incubation, cells were treated with different concentrations of recombinant LukS-PV protein, silver nanoparticles, and AgNPs + LukS-PV (100, 50, 25, 13, 6, 3, 2, and 1 μg/ml). After 24 and 48 h incubation, 10 μl of MTT reagent (10 mg/ml) was added to each well and incubated for 4 h at 37 °C in a humidified 5% CO_2_ atmosphere. Afterward, 100 μl of MTT solvent [included 10 ml SDS 10% and 10 μl HCL (12.5 M)] was added to each well to dissolve formazan crystals, and the mixture was incubated for 16 h in a cell culture incubator. The absorbance was read at 570 nm using an ELISA reader (Biorad, USA). All concentrations were examined three times. The half‐maximal inhibitory concentration (IC50) values were calculated.

### Cell ELISA

Cells were seeded in 96-well plates at a density of 1 × 10^4^ cells/well and incubated for 24 h at 37 °C in a humidified 5% CO_2_ atmosphere. In the next step, plates were washed once with PBS and 100 μl of 10% paraformaldehyde was added to each well and incubated for 15 min at room temperature. After washing with PBS, 200 μl of blocking solution (5% skim milk in PBS) was added to each well and incubated for 2 h at 37 °C. Then, cells were treated with different concentrations (100, 50, 25, 13, 6, 3, and 2 μg/ml) of recombinant LukS-PV protein, and recombinant LukS-PV protein-loaded silver nanoparticles and incubated for 1 h at 37 °C. After washing three times with PBS containing Tween-20 (PBS/T), 100 μl of HRP conjugated anti-His-tag antibody (1:2000) was added to each well and incubated for 2 h at 37 °C. After washing, o-Phenylenediamine substrate (OPD) (Sigma-Aldrich, Germany) was added to each well, and the reaction was stopped by adding 100 μl of sulfuric acid to each well. The absorbance was read at 492 nm using an ELISA reader (Biorad, USA).

### Apoptosis assay

Apoptosis was assessed via flow cytometry (FCM) by Annexin V-FITC/PI staining. For this purpose, cells were seeded into 12-well plates at a density of 1 × 10^5^ cells/ well. After 24 h incubation, cells were treated with concentration of IC50 and 2 X IC50 recombinant LukS-PV protein-loaded silver nanoparticles and incubated for 4 h at 37 °C in a humidified 5% CO_2_ atmosphere. Afterward, cells were collected and washed with cold PBS, and then the cells were resuspended in 500 ml Annexin V binding buffer, 5 ml Annexin V-FITC, and 5 ml PI, and then were incubated in the dark at room temperature for 30 min. All experiments were performed three times.

### Statistical analysis

Triplicates were performed for each method and the results were expressed as the mean ± standard deviation (SD). Statistical analysis between cancer and non-cancer cells and comparing mean values of IC50 between the two groups’ tests was performed through Student’s t-test using SPSS Statistics Version 26. p < 0.05 was considered statistically significant.

### Accession number

The accession number of the DNA sequence of the LukS-PV gene of *Staphylococcus aureus* strain HT20010734 was EU518761.1. The accession number of the optimized LukS-PV gene was OQ435276.

## Results

### Subcloning and expression of the recombinant LukS-PV protein

**Fig. 1:**
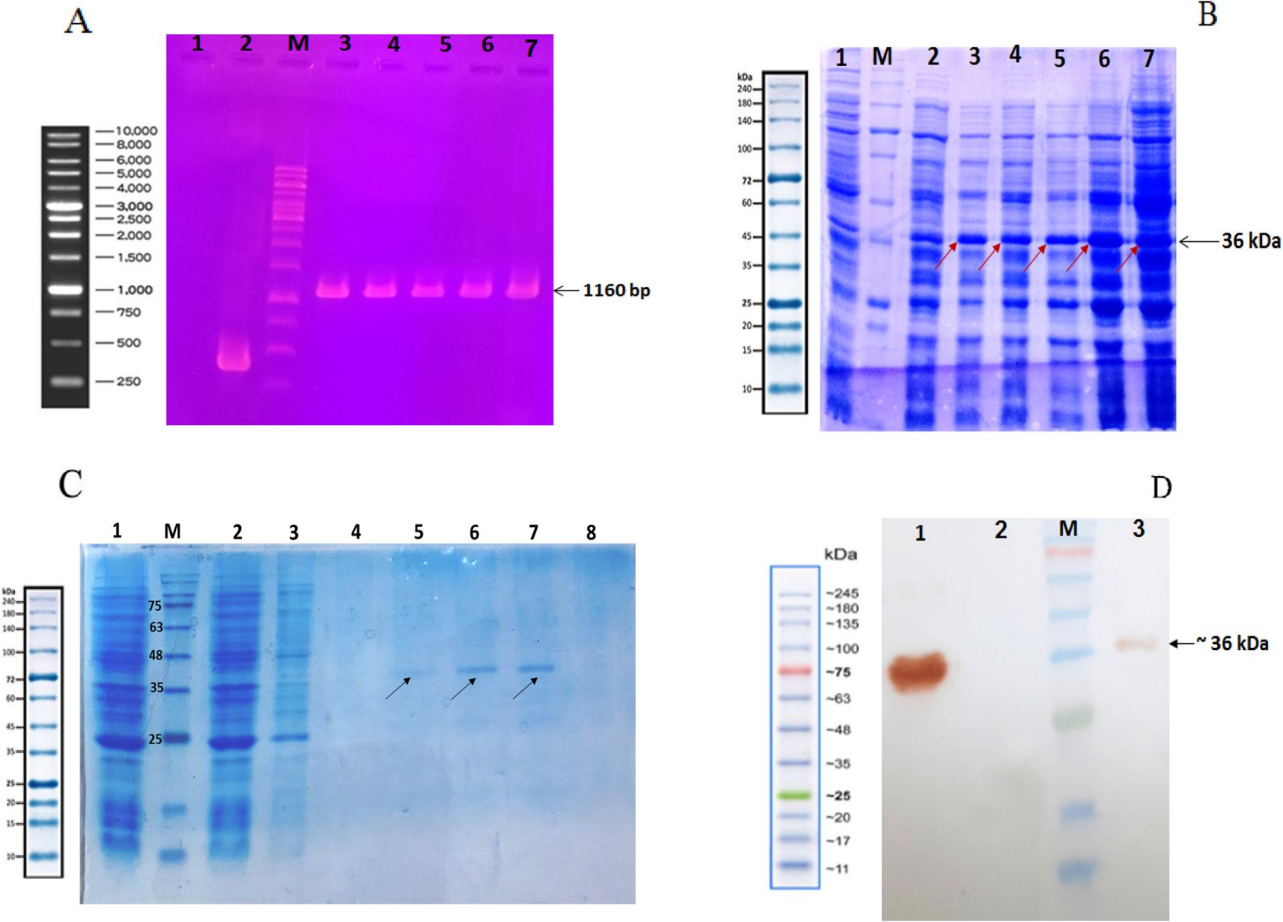
**a** Colony PCR to confirm the presence of LukS-PV gene in *E. coli* Rosetta (DE3) strain with T7 primers on 1% agarose gel. M: 1 kb DNA Ladder; (1) Negative control; (2) Positive control (empty pET28a); (3–7) Recombinant colonies. **b** Analysis of protein expression. M: Protein Marker; (1) Before induction sample; (2) induction sample after 4 h (clone 1); (3) induction sample after O/N (clone 1); (4) induction sample after 4 h (clone 2); (5) induction sample after O/N (clone 2); (6) induction sample after 4 h (clone 3); (7) induction sample after O/N (clone 3). **c** Protein purification using Ni–NTA affinity chromatography: M: Protein marker; (1) Lysate sample; (2) Ni–NTA flow through the sample; (3) Washing samples; (4–8) Eluted samples. **d** Analysis and confirmation of recombinant proteins purified in Rosetta (DE3) strain using Western blotting with anti-histidine antibody. M: Protein Marker; (1) Positive control (recombinant protein with His6-Tag); (2) Negative control; (3) Purified LukS-PV protein samples under denaturing conditions

The designed LukS-PV gene was synthesized after optimization. After the transformation of the PET 28a + LukS-PV constructs into the competent *E. coli* Rosetta (DE3) cells, screening of recombinant bacterial colonies was performed via colony PCR. The PCR product was analyzed by electrophoresis on 1% agarose gel and an 1160 bp was observed on the gel (Fig. 1a). The protein expression level was confirmed by the SDS-PAGE method and the band with a molecular weight of about 36 kDa was detected (Fig. 1b).

Purification of 6 × His-tagged recombinant LukS-PV proteins was done by Ni–NTA affinity column chromatography (Fig. 1c). The expressed recombinant protein was confirmed through the western blotting method using anti-His tag monoclonal antibody (Fig. 1d).

### Characterization of AgNPs before and after loading the recombinant LukS-PV protein

**Fig. 2:**
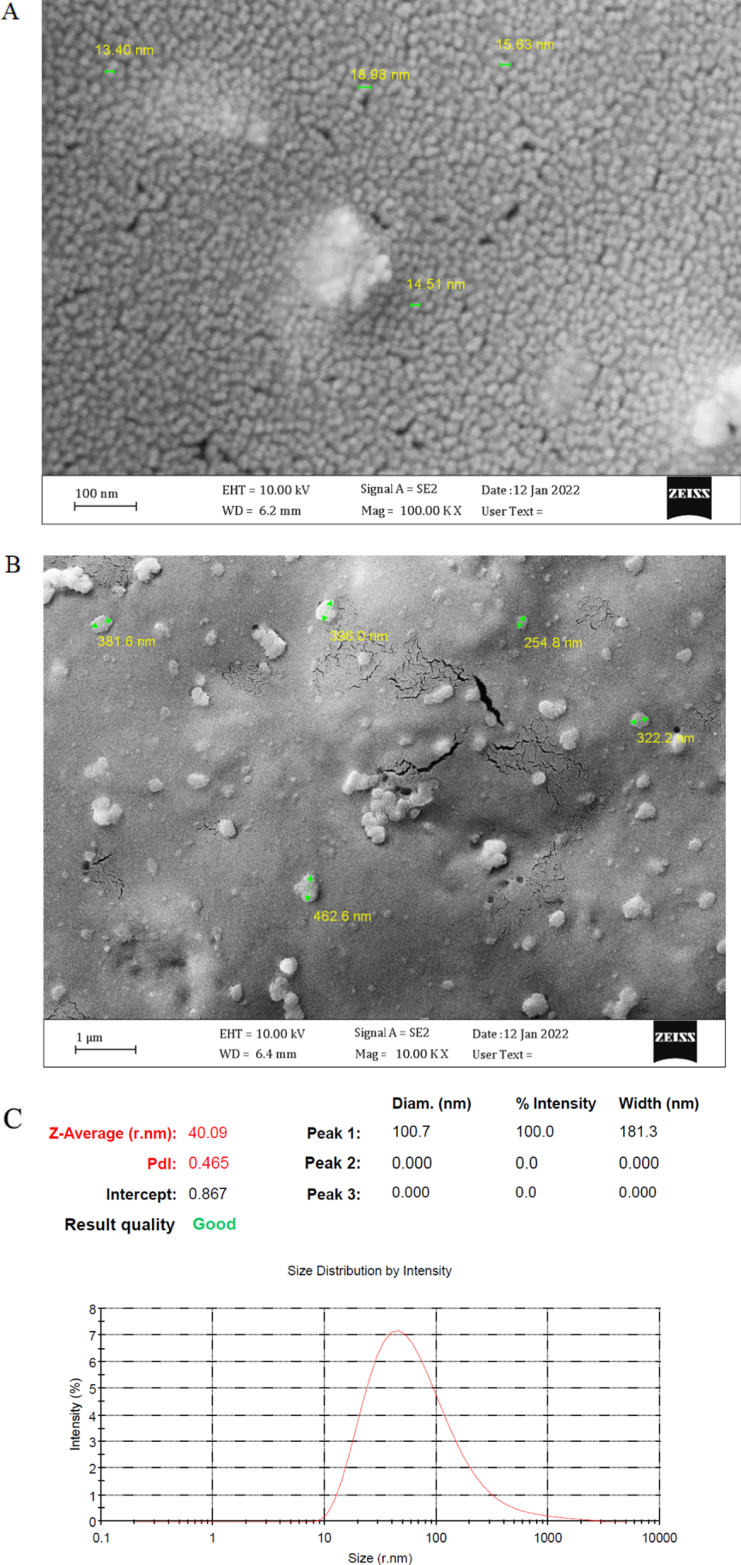

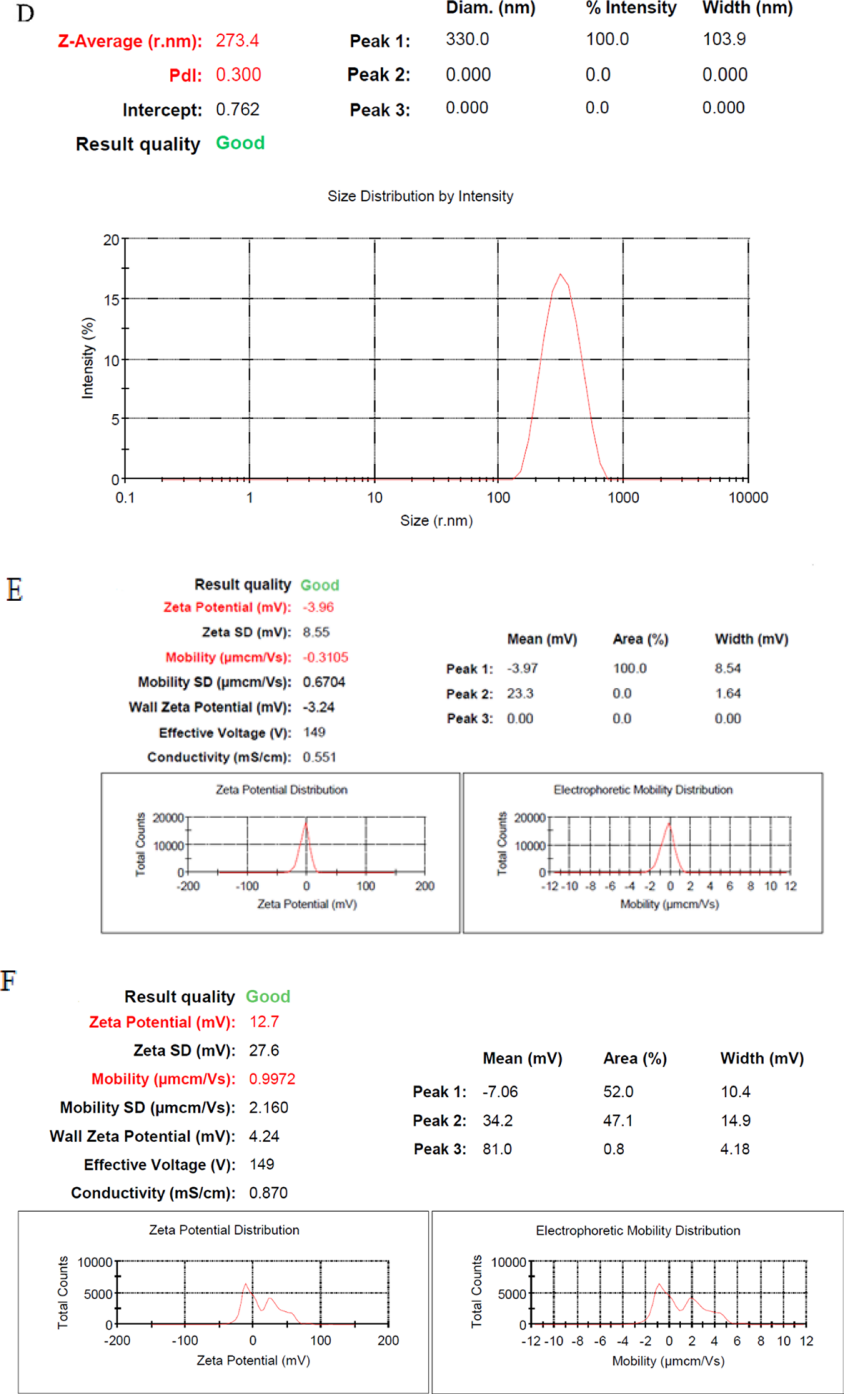
Field Emission Scanning Electron Microscope (FE-SEM) image of Ag NPs; **a** non-loaded Ag NPs, **b** recombinant LukS-PV protein-loaded Ag NPs. **c** The average particle size of Ag NPs before loading the recombinant LukS-PV protein. **d** The average particle size of Ag NPs after loading the recombinant LukS-PV protein. **e** Zeta potential distribution of Ag NPs before loading the recombinant LukS-PV protein. **f** Zeta potential distribution of Ag NPs after loading the recombinant LukS-PV protein

The nanoparticles morphology before and after loading recombinant LukS-PV protein was determined with FE-SEM images. The results revealed that the AgNPs before and after loading recombinant LukS-PV protein were spherical (Fig. 2a, b). Particle size and zeta potential of Ag NPs and Ag NPs after loading the recombinant LukS-PV protein were investigated using DLS. The average size and the polydispersity index (PDI) of Ag NPs were equal to 40.09 nm and 0.465, respectively and the quality of the obtained data was also desirable (Fig. 2c). The zeta potential value of Ag NPs was observed to be − 3.96 mV with conductivity of 0.551 (Fig. 2d). The average particles size and the PDI of Ag NPs after loading the recombinant LukS-PV protein were equal to 273.4 nm and 0.300, respectively and the quality of the obtained data was also desirable (Fig. 2e). The zeta potential value of Ag NPs after loading the recombinant LukS-PV protein was observed to be 12.7 mV with conductivity of 0.870 (Fig. 2f).

### Loading efficiency percent

Table. 1 shows the loading efficiency percent of different ratios of protein to silver nanoparticles. In ratios 1:1, the loading efficiency of protein to silver nanoparticles was 73.7%, in ratios 1:2, the loading efficiency of protein to silver nanoparticles was 85.2%, in ratios 1:3, the loading efficiency of protein to silver nanoparticles was 92.4% and, in ratios 1:4, the loading efficiency of protein to silver nanoparticles was 96.0%. The results showed that the highest amount of protein loading is done in a 1:4 ratio of protein to silver nanoparticles.

**Table 1:** The loading efficiency percent of protein-loaded silver nanoparticles

Different ratios	Loading efficiency (%)
1:1	73.7
1:2	85.2
1:3	92.4
1:4	96.0

### Determination of the recombinant LukS-PV protein release from silver nanoparticles

**Fig. 3:**
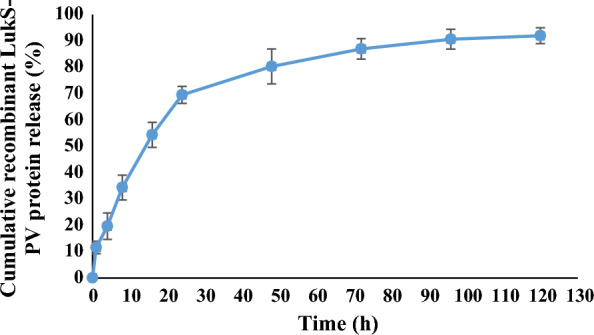
Cumulative release of recombinant LukS-PV protein

The results showed an accumulative release of 92% recombinant LukS-PV protein from silver nanoparticles in PBS for up to 5 days (Fig. 3). In the first 24 h, about 70% of the toxin was released, and in the next 24 h, there was a continuous release of toxin. These outcomes indicate that the toxin release cycle consists of two phases: an initial blast phase and a constant phase.

### Effect of recombinant LukS-PV protein on cytotoxicity

**Fig. 4:**
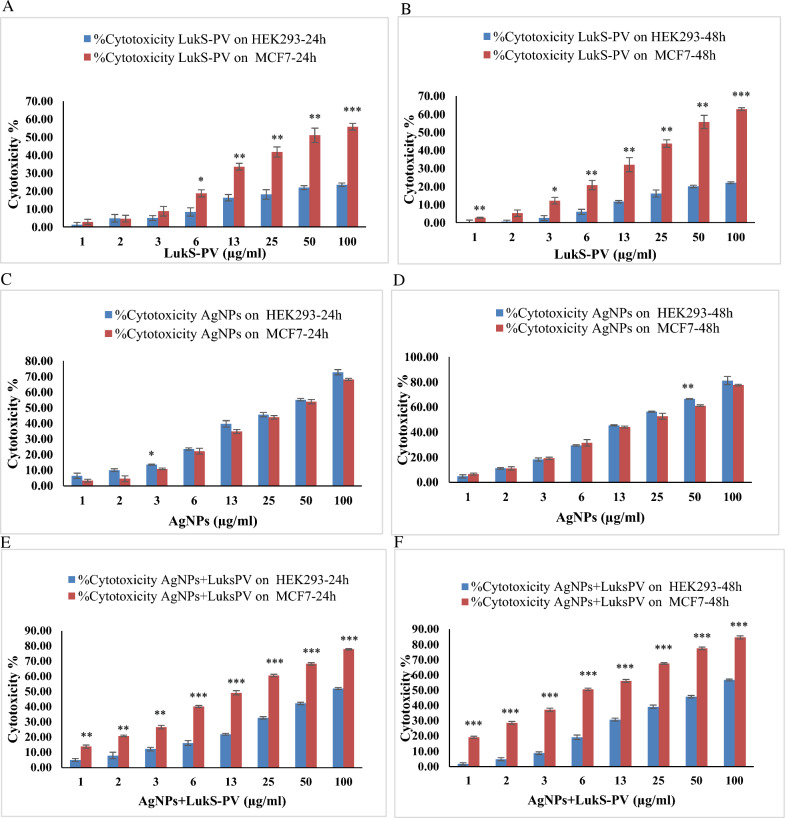
Cytotoxicity was measured using an MTT assay. **a, b** MCF7 cells and HEK293 cells were treated with different concentrations of recombinant LukS-PV protein for 24 h (**a**) and 48 h (**b**). **c, d** MCF7 cells and HEK293 cells were treated with different concentrations of chemically synthesized Ag NPs for 24 h (**c**) and 48 h (**d**). **e, f** MCF7 cells and HEK293 cells were treated with different concentrations of AgNPs + LukS-PV for 24 h (**e**) and 48 h (**f**). *p < 0.05; **p < 0.01; ***p < 0.001 versus HEK293

The cytotoxic effect of recombinant LukS-PV protein with different concentrations in MCF7 and HEK293 cells is shown in Fig. 4a, b. Recombinant LukS-PV protein had a more effective cytotoxic effect in MCF7 cells than HEK293 cells at all concentrations except concentration 2 at 24 h. The cytotoxic effect of LukS-PV was significantly different (p < 0.05–p < 0.001) in all concentrations except 1, 2, and 3 at 24 h and concentration 2 at 48 h.

### Effect of chemically synthesized Ag NPs on cytotoxicity

The MTT results indicated that the cytotoxicity effect of silver nanoparticles in some concentrations was even higher in HEK293 than in MCF7 cells (Fig. 4c, d). Statistical analysis of the t-test did not show a significant difference (P > 0.05) in the cytotoxic effect of these nanoparticles between the two cell lines except for the concentration of 3 in 24 h and 50 in 48 h (p < 0.05 and p < 0.01 respectively).

### Effect of AgNPs + LukS-PV on cytotoxicity

To assess the effect of AgNPs + LukS-PV on cytotoxicity, MCF7 and, HEK293 cells were cultured with different concentrations of AgNPs + LukS-PV for 24 and 48 h. MTT assays suggested that AgNPs + LukS-PV treatment increases the cytotoxicity of MCF7 cells in a dose- and time-dependent manner. The inhibition of MCF7 cells after 24 and 48 h incubation with various concentrations of AgNPs + LukS-PV increased considerably. There was a considerable difference between the cytotoxicity effect of AgNPs + LukS-PV at all concentrations in MCF7 and HEK293 cells (Fig. 4e, f). The average cytotoxicity effect of AgNPs + LukS-PV in the concentrations tested in MCF7 was significantly higher than that of HEK293 (p < 0.05–p < 0.001).

IC50 values are listed in Table 2. The results showed a significantly lower value of the IC50 of the AgNPs + LukS-PV in comparison with the LukS-PV against MCF7 and HEK293 cell lines (p < 0.05–p < 0.001).

**Table 2:** IC50 of LukS-PV and AgNPs + LukS-PV after incubation with MCF7 and HEK293 cells for 24 h and 48 h

Treatment cell lines	LukS-PV	AgNPs + LukS-PV	P value^†^
MCF7 24 h	49.64 ± 7.064	13.11 ± 0.699	0.012*
MCF7 48 h	45.35 ± 5.381	6.17 ± 0.171	0.000*
HEK293 24 h	213.56 ± 14.620	96.18 ± 2.027	0.004*
HEK293 48 h	226.95 ± 8.409	88.18 ± 1.645	0.001*

^†^Student’s t-test

*Significant difference

### Comparison effect of AgNPs + LukS-PV and LukS-PV on cytotoxicity

**Fig. 5:**
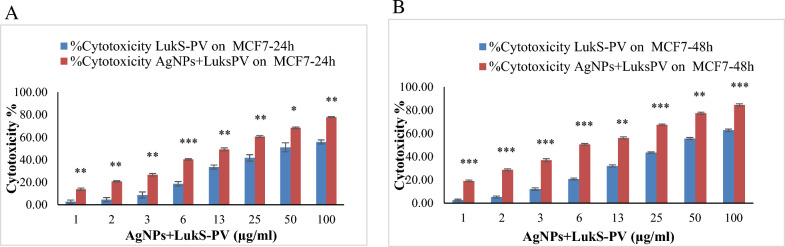
Comparison of cytotoxicity of AgNPs + LukS-PV with LukS-PV in different concentrations at 24 h (**a**) and 48 h (**b**). *p < 0.05; **p < 0.01; ***p < 0.001

Considering that the cytotoxic effect of AgNPs in some concentrations was even higher in HEK293 than in MCF7 cells, the comparison was made only between AgNPs + LukS-PV and LukS-PV. As seen in Fig. 5, the cytotoxic effect of AgNPs + LukS-PV in all concentrations is significantly higher than that of LukS-PV in the cancer group (p < 0.05–p < 0.001).

### Examining the binding of recombinant LukS-PV protein and AgNPs + LukS-PV to the cell by cell ELISA

**Fig. 6:**
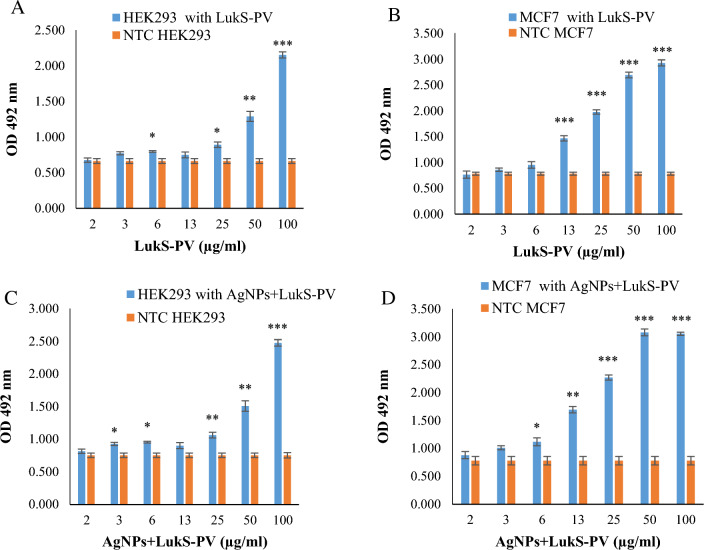
Results of Cell ELISA. **a** HEK293 cells treated with different concentrations of recombinant LukS-PV protein and NTC. **b** MCF7 cells and NTC treated with different concentrations of recombinant LukS-PV protein. **c** HEK293 cells and NTC treated with different concentrations of recombinant AgNPs + LukS-PV. **d** MCF7 cells and NTC treated with different concentrations of AgNPs + LukS-PV. *p < 0.05; **p < 0.01; ***p < 0.001 versus NTC

Cell ELISA was used to investigate the binding of the recombinant LukS-PV protein and AgNPs + LukS-PV to the cell. OD difference was observed between the non-treated cells (NTC) and treated cells for their interaction with MCF7 cells and HEK293 cells (Fig. 6). This OD difference indicates the binding or entry of recombinant LukS-PV protein and AgNPs + LukS-PV to MCF7 and HEK293 cells.

### AgNPs + LukS-PV induces apoptosis

**Fig. 7:**
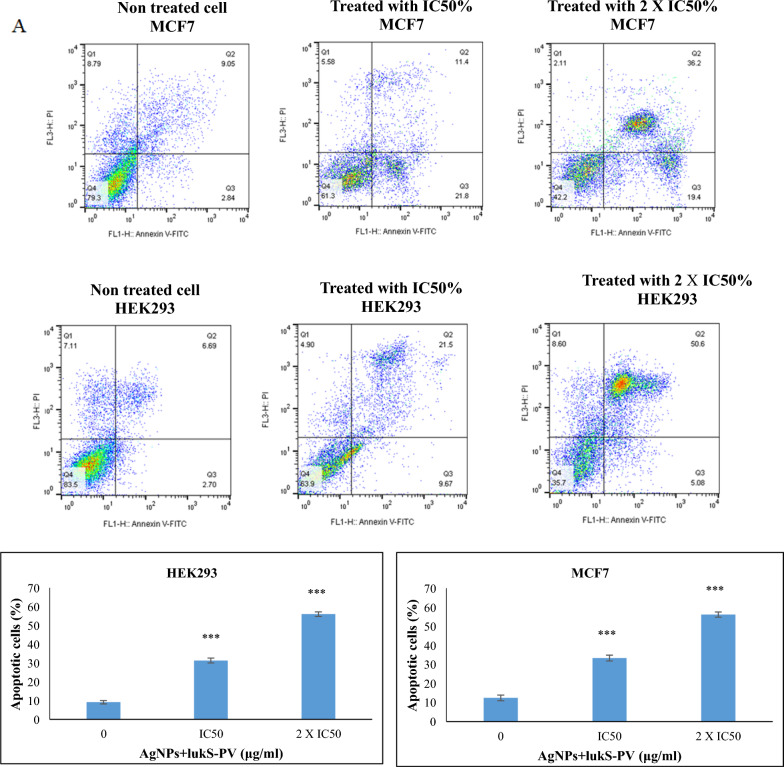
Cells were stained with Annexin V-FITC/PI, and the ratio of apoptosis was analyzed by FCM (**a, b**) MCF7 cells, and HEK293 cells were treated with different concentrations of AgNPs + LukS-PV for 24 h. *p < 0.05; **p < 0.01; ***p < 0.001 versus control

Detection of apoptotic cells using flow cytometry displayed that AgNPs + LukS-PV inhibited the proliferation of MCF7 cells by an increase in the frequency of apoptotic cells. As shown in Fig. 7, in MCF7 cells the frequency of apoptotic cells was 33.2% and 55.6% upon treatment with AgNPs + LukS-PV in IC50 and 2 X IC50 concentrations, respectively, while in HEK293 cells the frequency of apoptotic cells was 31.17% and 55.68% at its own IC50 and 2 X IC50, respectively.

## Discussion

This study is the first evaluation of an AgNPs + LukS-PV comprised of chemically synthesized AgNPs loaded with LukS-PV protein as a potential candidate for breast cancer treatment. Also, the effect of AgNPs + LukS-PV on MCF7 cells and HEK293 cells has been compared with the recombinant LukS-PV protein and chemically synthesized silver nanoparticles.

The standard treatment for breast cancer is chemotherapy. In chemotherapy, anti-cancer drugs target the cancer cells or tissues by inducing cytotoxicity or revving specific immune responses to eliminate the cancerous tissues. This drug can damage normal cells as well as cancer cells, and cause various side effects. Therefore, it is necessary that drugs specifically target and kill harmful or cancerous cells (Lee et al. 2020; Hussein and Abdullah 2021). To this end, nanotechnologies with various advantages have been applied for the drug delivery of anticancer drugs, including penetrating tissues, improving the targeting and diffusion of the drug, help the possible release of drugs to the target site. Anticancer drugs have stability and solubility problems, nanoparticles as drug delivery systems could overcome these problems, keeping the drug from enzymatic degradation and thereby extending the the drug half-life within the body (Navya et al. 2019; Hussein and Abdullah 2021; Sim and Wong 2021). According to reports, bacterial toxins have specific cytotoxic impacts on tumor cells, and they have obtained increasing attention in the development of new anticancer drugs (Xu et al. 2021).

The potential cytotoxicity of AgNPs + LukS-PV was tested by MTT assay and showed that AgNPs + LukS-PV can be significantly toxic (p < 0.05–p < 0.001) to the MCF7 cells. Although the cytotoxicity effect of AgNPs + LukS-PV has also been shown in normal cells, in all concentrations, the cytotoxicity effect of AgNPs + LukS-PV was significantly (p < 0.05–p < 0.001) higher in cancer cells than in normal cells. Also, the results showed that the recombinant LukS-PV protein has intense cytotoxicity in MCF7 cells whereas it has little impact on normal HEK293 cells. Although the cytotoxicity effect of recombinant LukS-PV protein has also been shown in normal cells, in most concentrations, the cytotoxicity effect of recombinant LukS-PV protein was higher in cancer cells than in normal cells. The cytotoxic effect of LukS-PV was significantly different (p < 0.05–p < 0.001) in all concentrations except 1, 2, and 3 at 24 h and concentration 2 at 48 h. Sun et al. found that LukS-PV induces THP-1 cell differentiation and apoptosis by downregulating NF1 and BCL2 (Sun et al. 2017). Dai et al. found that LukS-PV induces differentiation by activating the ERK signaling pathway and c-JUN/c-FOS in human acute myeloid leukemia cells (Dai et al. 2016). Additionally, LukS-PV induces apoptosis in AML cells by targeting the C5a receptor and inhibits AML cell proliferation in vitro and in vivo, and has no noticeable side effects on mice. (Zhang et al. 2019; Shan et al. 2015; Xu et al. 2020). Wang et al. demonstrated that LukS-PV inhibited the proliferation and induced apoptosis in hepatocellular carcinoma (HCC) cells by downregulating histone acetylation (Wang et al. 2020).

Heretofore multiple studies have been performed to examine the anticancerous impact of different metallic nanoparticles on breast cancer cell lines (Subhan and Muzibur Rahman 2022). Among other metallic nanoparticles, AgNPs are preferable due to their specific characteristics, such as being less toxic and having high anticancer activities (Alves et al. 2022). The current study demonstrated that chemically synthesized AgNPs possessed anticancer activity on MCF7 and non-cancerous HEK293 cell lines. Obtained results indicated that the chemically synthesized AgNPs could not selectively kill tumor cells. AgNPs had no significant difference in most concentrations, and even in most concentrations, they had a higher cytotoxicity effect on normal cell lines. Nevertheless, previous research demonstrated the high cytotoxic impact of AgNPs on HeLa, HepG2, and A549 cells (Venugopal et al. 2017; Alahmad et al. 2021). Based on the results of studies, chemically synthesized AgNPs had anticancer activity against the MCF7 and AU565 cells (Dinparvar et al. 2020).

In the present study, recombinant LukS-PV protein was loaded to AgNPs, and according to Fig. 5, it had a better effect. The cytotoxic effect of AgNPs + LukS-PV on cancer cells was significantly higher than LukS-PV at all concentrations.

As demonstrated in Table 2, the loading of recombinant LukS-PV protein on AgNPs raised its anticancer activity in MCF7 cells and had a lesser effect on normal HEK293 cells. The higher anticancer activity of AgNPs + LukS-PV is represented in the lower value of the IC50 compared to LukS-PV. Although the cytotoxicity of the AgNPs + LukS-PV in cancer cells is significantly higher than normal cells, the cytotoxicity of higher doses is more than lower doses in normal cells. Nanoparticles as drug delivery systems have attracted considerable attention due to targeting the diseased area with controlled drug release (Patra et al. 2018). Silver nanoparticles due to their intrinsic properties, including their stability, high drug loading capacity, ability to bind a wide range of organic molecules, strong absorption virtues, controlled drug delivery, and low toxicity act as drug delivery systems (Bahrami et al. 2017; Burdușel et al. 2018; Ivanova et al. 2018). AgNPs can serve as drug delivery agents and have displayed anticancer activity against several cancer cell lines (Hussein and Abdullah 2021). Several research groups demonstrated that the incorporation of the drug with the AgNPs has the cytotoxic actions considerably enhanced (Benyettou et al. 2015; Hussein and Abdullah 2021). The increase in the anticancer activity of recombinant LukS-PV protein after their loading of AgNPs could be due to AgNPs’ ability to enter more cells as drug carriers and deliver LukS-PV with them. Currently, immunotoxins have been used as a cancer therapy strategy (Keshtvarz et al. 2021). One of the advantages of nano toxins over immunotoxins is that due to their small size, nanoparticles are not antigenic or immunogenic (Gholami et al. 2020).

Flow cytometry was performed based on the IC50 concentration of each of the HEK293 and MCF7 lines. Although the frequency of apoptotic cells in the concentration of IC50 in HEK293 and MCF7 is almost similar, the concentration causing IC50 is significantly (p < 0.001) higher in normal cells.

The data presented in this article indicates that AgNPs + LukS-PV could induce apoptosis of MCF7 cells at low concentrations (13 μg/ml after 24), but induce apoptosis of HEK293 cells at high concentrations (96 μg/ml after 24). Previous studies indicated that the mitochondrial pathway was involved in the apoptosis process induced by LukS-PV. LukS-PV induces apoptosis through the activation of caspase-8, caspase-9, and caspase-3, and increases levels of pro-apoptotic protein Bax while decreasing levels of anti-apoptotic protein Bcl-2 (Wang et al. 2020; Qiang et al. 2020; Zhang et al. 2019; Bu et al. 2013). Zhang et al. demonstrated that nanomaterials with drugs prompt cancerous cell apoptosis in vitro and in vivo. Also, they indicated that nanomaterials with drugs enhanced cell death rate, and enhanced inhibition of tumor growth by affecting caspase, NF-κB, and Bcl-2 expression (Zhang et al. 2013). Perhaps AgNPs + LukS-PV stimulate cancer cell apoptosis through the mitochondrial pathway in MCF7 cells.

In conclusion, according to the results of this study, AgNPs + LukS-PV cannot be considered as a novel strategy for cancer therapy. However, further studies are needed to compare the effect of AgNPs + LukS-PV and LukS-PV with other available and proposed options on healthy cells to choose a more effective and less dangerous option.

## Data Availability

The datasets generated and analyzed in this study and all materials are available on request from the corresponding author.
